# Metadherin promotes metastasis by supporting putative cancer stem cell properties and epithelial plasticity in pancreatic cancer

**DOI:** 10.18632/oncotarget.19802

**Published:** 2017-08-02

**Authors:** Kensuke Suzuki, Shigetsugu Takano, Hideyuki Yoshitomi, Hitoe Nishino, Shingo Kagawa, Hiroaki Shimizu, Katsunori Furukawa, Masaru Miyazaki, Masayuki Ohtsuka

**Affiliations:** ^1^ Department of General Surgery, Graduate School of Medicine, Chiba University, Chiba, Japan

**Keywords:** metadherin, pancreatic cancer, cancer stem cell, epithelial plasticity, MET

## Abstract

Pancreatic ductal adenocarcinoma (PDAC) has a high metastatic potential. However, the mechanism of metastatic colonization in PDAC remains poorly understood. Metadherin (MTDH) has emerged in recent years as a crucial mediator of metastasis in several cancer types, although the biological role of MTDH in PDAC has not been investigated. Here, we demonstrated the functional roles of MTDH in PDAC progression, especially focusing on the metastatic cascade. *In vitro* studies showed that MTDH provides cancer stem cell (CSC) properties in metastatic PDAC cells and contributes to anoikis resistance with epithelial characteristics in PDAC cells. We also performed *in vivo* studies using both orthotopic transplantation and intra-portal vein injection as experimental models of liver metastasis to examine the function of MTDH at the metastatic site. MTDH knockdown dramatically reduced the incidence of liver metastases along with epithelial features in both experimental mouse models. Collectively, MTDH facilitates metastatic colonization with putative CSC and epithelial properties in PDAC cells. PDAC cells were transiently treated with TGF-β1 to investigate the roles of MTDH on epithelial plasticity. Intriguingly, MTDH expression was negatively correlated with Twist1 expression during the Mesenchymal-Epithelial transition (MET) induction in metastatic PDAC cells. These results suggest that MTDH may contribute to MET induction via downregulation of Twsit1. Lastly, immunohistochemistry indicated that MTDH overexpression is closely associated with hematogenous metastasis and predicts poor prognosis in patients with PDAC. This is the first demonstration of MTDH function in PDAC metastatic colonization. Our data suggest that MTDH targeting therapy could be applied to control PDAC metastasis.

## INTRODUCTION

Pancreatic ductal adenocarcinoma (PDAC) is one of the most lethal cancers and is the fourth most-frequent tumor-related cause of death in the Western world [1]. Surgical resection is the only hope for curative treatment for PDAC [2]. However, 50% of patients with PDAC are diagnosed with unresectable disease with distant metastasis and about 70% of the patients who undergo radical surgical resection develop metastatic recurrence [3]. These data suggest the existence of distant micrometastasis even at early stage of carcinogenesis in PDAC. Indeed, Rhim *et al.* showed that circulating pancreatic cells from PanIN mice are seeded in the liver using a genetically engineered mouse model [4]. Therefore, especially in PDAC, it is of great clinical value to elucidate the mechanism underlying the outgrowth of disseminated cancer cells into macroscopic metastases.

Numerous studies described that the activation of the Epithelial-Mesenchymal transition (EMT) program confers cancer stem cell (CSC) properties, and these are responsible for tumorigenesis and metastasis [5, 6]. In contrast, emerging evidence suggests that the absence of Snail1 or Twist1, master regulators of EMT, does not alter cancer progression on the capacity for local invasion and metastasis to the liver or lung in genetically engineered mouse models of PDAC [7]. In line with this, recent studies indicated that the reversion of EMT is essential for disseminated tumor cells to proliferate and form metastases [8]. Additionally, the deactivation of Twist1 induces a mesenchymal-epithelial transition (MET) and stem-like phenotype at the metastatic site in breast cancer [8]. Thus, understanding the underlying mechanisms of EMT/MET is important to developing novel therapeutic approaches to target the metastatic cascade.

Metadherin (MTDH), also called AEG1 or LYRIC/3D3, is a single-pass transmembrane protein encoded by a gene located on chromosome 8q22 [9]. MTDH (AEG-1) was originally cloned as a human immunodeficiency virus-1 (HIV-1)-inducible gene in primary human fetal astrocytes [10], and MTDH contributes to cell proliferation in embryogenesis [11]. In the field of oncology, MTDH was initially identified as a regulator for metastasis in breast cancer cells [12]. High MTDH expression is associated with poor prognosis in a large spectrum of cancer types [13, 14]. Functionally, Dr. Kang’s group recently demonstrated that the interaction of MTDH and Staphylococcal nuclease domain-containing 1 is crucial for expansion and activity of tumor-initiating cells in diverse oncogene- and carcinogen-induced mammary tumors [15]. However, the functional roles of MTDH in PDAC progression, especially during the metastatic cascade, are poorly understood.

In this study, we focused on the functional contribution of MTDH to metastasis and undergoing epithelial plasticity, involving putative CSC functions in PDAC progression. MTDH regulation provides novel insights on the governance of EMT and MET in primary and metastatic PDAC and a new platform for translational therapeutics.

## RESULTS

### MTDH is overexpressed in metastatic PDAC cells

**Figure 1:**
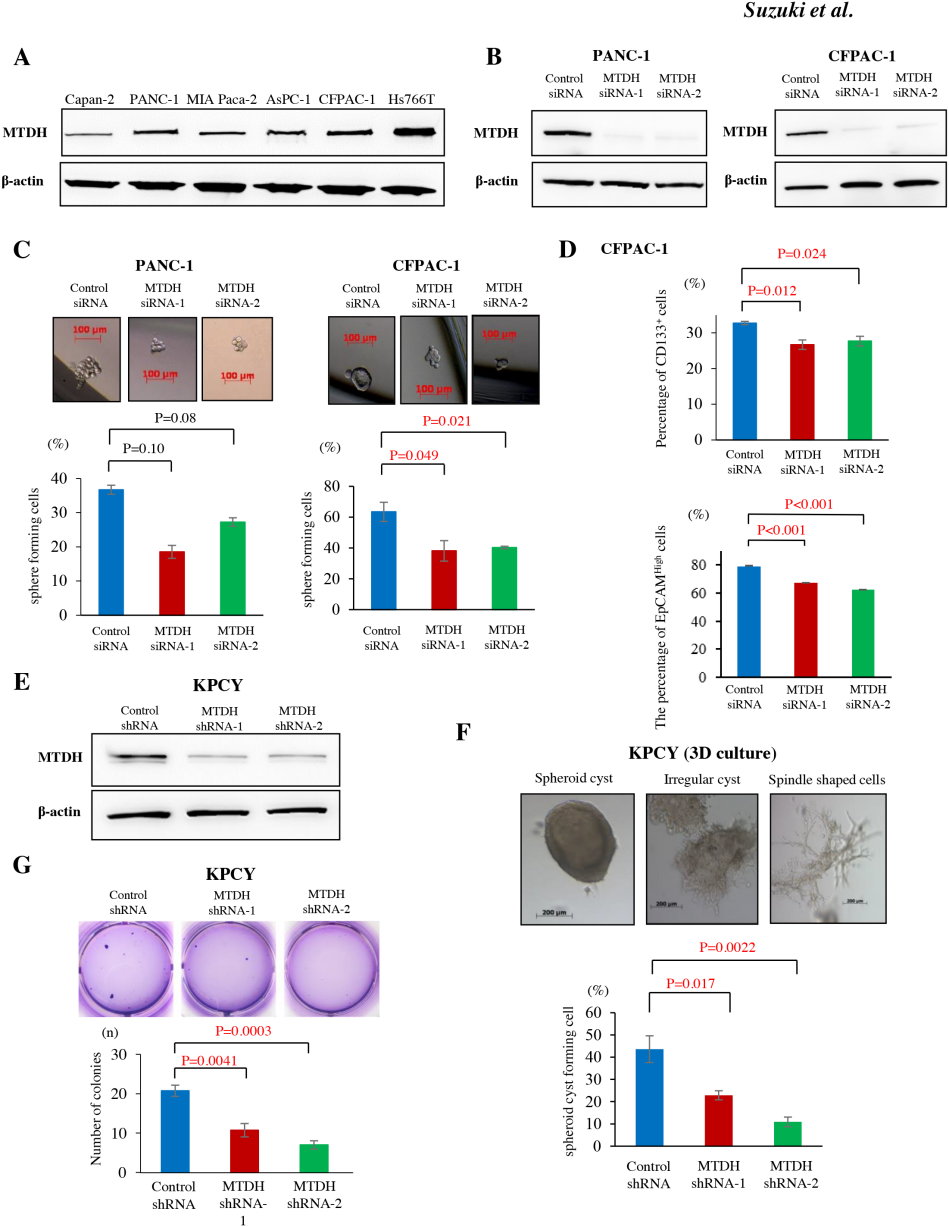
MTDH expression is associated with stem cell like property in metastatic PDAC cells and correlates with anoikis resistance with epithelial property in KPCY cells **(A)** MTDH protein expression in human pancreatic cell lines was analyzed by western blot. Metastatic PDAC cell lines (CFPAC-1 and Hs766T) showed higher levels of MTDH compared to primary invasive PDAC cell lines (Capan-2, PANC-1, and MIA Paca-2). **(B)** MTDH knockdown in PANC-1 and CFPAC-1 cells by MTDH siRNA-1 and -2 was confirmed by western blot analysis. **(C)** The sphere formation rate in PANC-1 and CFPAC-1 cells treated with negative control siRNA, MTDH siRNA-1 or -2. Upper panel: Representative sphere forming cells of CFPAC-1 cells in pancreatosphere formation assays. Sphere cells in CFPAC-1 cells treated with control siRNA (left panel), MTDH siRNA-1 (middle panel), and MTDH siRNA-2 (right panel). Lower panel: MTDH knockdown significantly decreased the sphere formation rate in CFPAC-1 cells, but not in PANC-1 cells (student’s t-test). **(D)** CD133 expression and EpCAM expression in CFPAC-1 were analyzed by flow cytometry. The proportions of CD133^+^ cells and EpCAM^High^ cells were significantly decreased by MTDH knockdown in CFPAC-1 cells (student’s t-test). Values are shown as mean ± SEM. **(E)** MTDH knockdown by MTDH shRNA-1 and -2 was confirmed by western blot analysis. **(F)** Upper panel: Various morphological changes of KPCY cells in 3D organotypic cell culture. Lower panel: MTDH knockdown significantly decreases the ratio of spheroid cyst among KPCY cells treating with shRNAs. **(G)** The number of colonies was significantly reduced by MTDH knockdown in KPCY cells (student’s t-test).

At first, we investigated the level of *MTDH* mRNA and protein expression in PDAC cell lines. Western blot analyses showed that MTDH was highly expressed in PDAC cell lines, especially in the metastatic cell lines (CFPAC-1; liver metastatic cells, Hs766T; lymph node metastatic cells) (Figure 1A). Similarly quantitative RT-PCR data showed that *MTDH* mRNA levels in these metastatic PDAC cell lines were high compared to that of primary PDAC cell lines (Supplementary Figure 1A). Furthermore, we confirmed that MTDH protein expression in mouse liver metastatic PDAC cells is higher than that in mouse primary PDAC cells (Supplementary Figure 1B). These results implicated that MTDH might be associated with metastasis in PDAC.

### MTDH induces cancer stem cell-like property in metastatic PDAC cells

Recent studies demonstrated that CSC properties might play a crucial role in cancerous metastatic progression. To confirm the correlation between MTDH and CSC properties in *in vitro*, pancreatosphere formation assay was performed in both human and murine PDAC cell lines. Using MTDH specific siRNAs (Figure 1B), MTDH knockdown resulted in the significant decrease of sphere forming cells in CFPAC-1, liver metastatic PDAC cells (Figure 1C). Consistently, the number of sphere forming cells was significantly decreased in KPC1Liv, mouse liver metastatic PDAC cells by MTDH knockdown (Supplementary Figure 1C and 1D). We next examined whether MTDH is related to the expression of cell surface markers, CD133 or Epithelial cell adhesion molecule (EpCAM), a representative CSC marker in PDAC. For this purpose, we analyzed CD133 expression and EpCAM expression in CFPAC-1 by flow cytometry. MTDH knockdown by the two specific siRNAs significantly decreased the proportions of the CD133^+^ cells and EpCAM^High^ cells compared with cells treated with control siRNA (Figure 1D). Additionally, we observed that the proportion of the CD133^+^ cells in MTDH knockdown cells significantly decreased compared with control cells in KPC1Liv cells (Supplementary Figure 1E). Taken together, these results suggested that MTDH is associated with CSCs properties in metastatic PDAC cells *in vitro*.

### MTDH contributes to anoikis resistance and supports epithelial properties in PDAC cells

Based on the association between MTDH and metastasis, we hypothesized that MTDH might prevent cancer cells from anoikis after extravasation in metastatic site. To clarify this hypothesis both *in vitro* and *in vivo*, we attempted MTDH knockdown using two short hairpins targeting MTDH (MTDH shRNA-1 and MTDH shRNA-2) in KPCY cells, lineage YFP-labeled cancer cells from pancreatic tumor-bearing KPCY mice. After confirming MTDH knockdown efficiency in these KPCY-MTDH shRNA-1 and -2 cells (Figure 1E), we first performed three-dimensional (3D) cell culture to investigate whether MTDH influences of the morphological of PDAC cells. KPCY control cells expressing MTDH formed significantly more spheroid cysts compared to MTDH knockdown cells (Figure 1F). The morphology of MTDH-knocked down cells was altered, exhibiting mesenchymal spindle shaped cells in 3D cell culture. These results suggested that MTDH plays a functional role in maintaining the epithelial phenotype morphologically in PDAC cells.

Next, anoikis assay was performed to evaluate the potential role of MTDH in resistance to apoptosis after losing contact from extracellular matrix (Supplementary Figure 1F). Compared to that of control KPCY cells, the resistance of MTDH-knocked down KPCY cells to anoikis was significantly decreased (Figure 1G). These data suggest that MTDH knockdown leads to functionally impaired anchorage-independent growth *in vitro*.

### MTDH promotes PDAC liver metastasis in *in vivo* models

**Figure 2:**
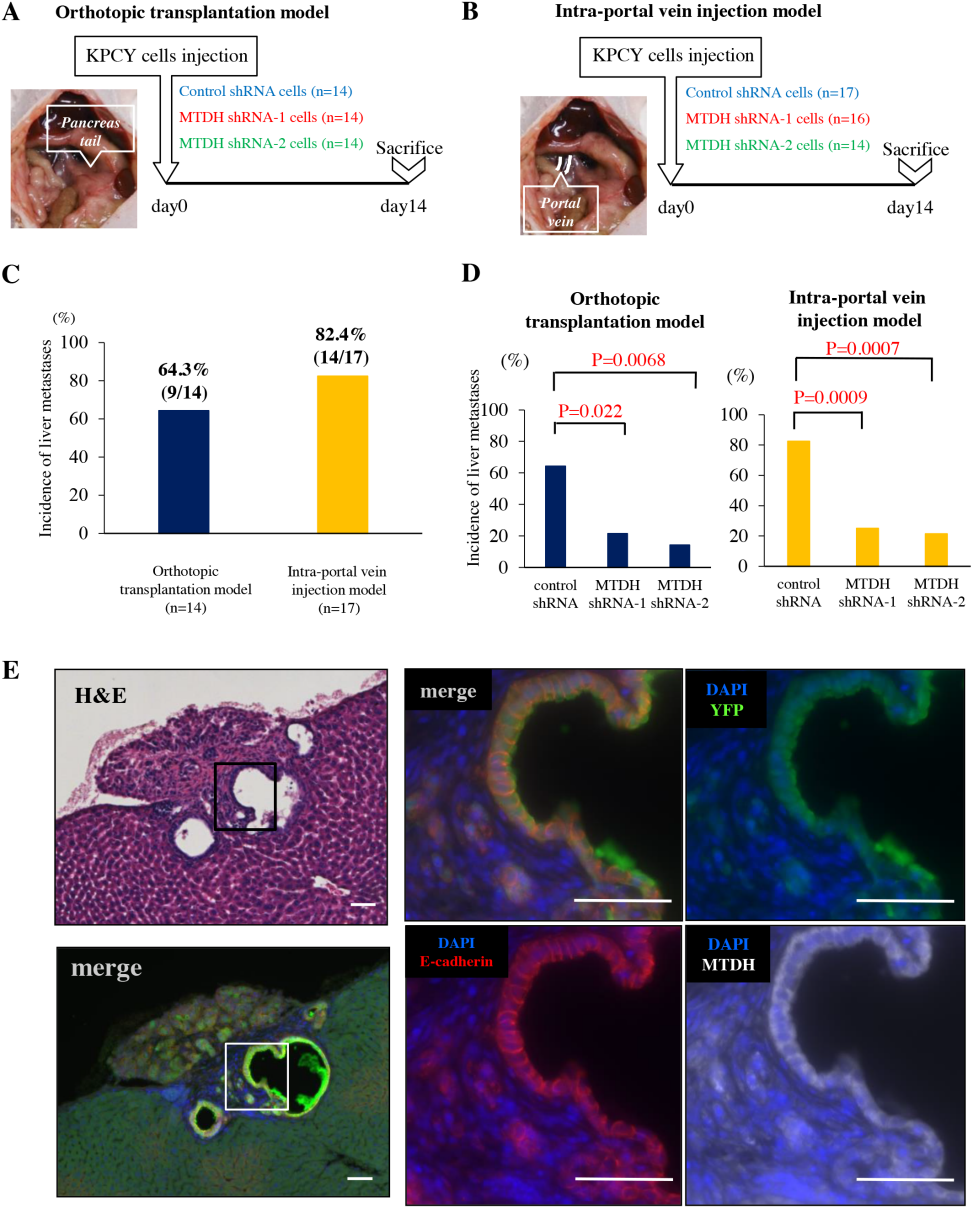
MTDH knockdown decreased the incidence of liver metastases in experimental mouse PDAC models **(A)** Experimental design for orthotopic transplantation of KPCY cells transduced with control shRNA (n=14), MTDH shRNA-1 (n=14), or shRNA-2 (n=14). **(B)** Experimental design for intra-portal vein injection of KPCY cells transduced with control shRNA (n=17), MTDH shRNA-1 (n=16), or shRNA-2 (n=14). **(C)** Difference in the incidence of liver metastasis between orthotopic transplantation model (left panel) and intra-portal vein injection model (right panel). **(D)** The liver metastasis formation rate of KPCY cells transduced with either control shRNA, MTDH shRNA-1, or shRNA-2 in both orthotopic transplantation model and intra-portal vein injection model. MTDH knockdown significantly decreases the incidence of liver metastases in these two models. **(E)** Quadruple immunofluorescence staining for YFP (green), MTDH (white), E-cadherin (red) and DAPI (blue) in metastatic colonies of liver tissue. MTDH and E-cadherin are co-expressed in liver metastases of PDAC. Bar, 50μm.

Considering that MTDH promotes CSC properties and anoikis resistance and supports epithelial characteristics in PDAC cells, we next examine whether MTDH facilitates metastatic colonization *in vivo*. To this end, we designed two *in vivo* experiments using KPCY cells. We first performed orthotopic transplantation in which three kinds of KPCY cells transducing with control shRNA, MTDH shRNA-1 and shRNA-2 were injected into the tail of pancreas of nude mice (Figure 2A). Primary tumor volumes of KPCY-MTDH shRNA-1 and shRNA-2 cells were significantly smaller than that of tumors obtained with KPCY-Control shRNA cells (Supplementary Figure 2).

To avoid the influence on primary tumor volume and focus on the ability of metastatic colonization, we utilized an *in vivo* liver metastasis assay in which KPCY cells are injected directly into the portal vein of mice, thereby bypassing invasion and intravasation at the primary tumor site (Figure 2B). Indeed, only 9/14 mice (64.3%) presented liver metastasis in orthotopic transplantation model, whereas 14/17 mice (82.4%) showed metastasis to the liver in the intra-portal vein injection model (Figure 2C). Importantly, MTDH knockdown significantly decreased the frequency of liver metastases in both experimental models (Figure 2D). Additionally, triple immunofluorescence staining for YFP, MTDH, E-cadherin exhibited that MTDH is highly co-expressed with E-cadherin in overt metastatic colonies of liver tissues (Figure 2E). These results clearly demonstrated that MTDH contributes to metastatic colonization along with epithelial features during PDAC progression.

### MTDH facilitates MET by suppressing Twist1 in metastatic PDAC cells

**Figure 3:**
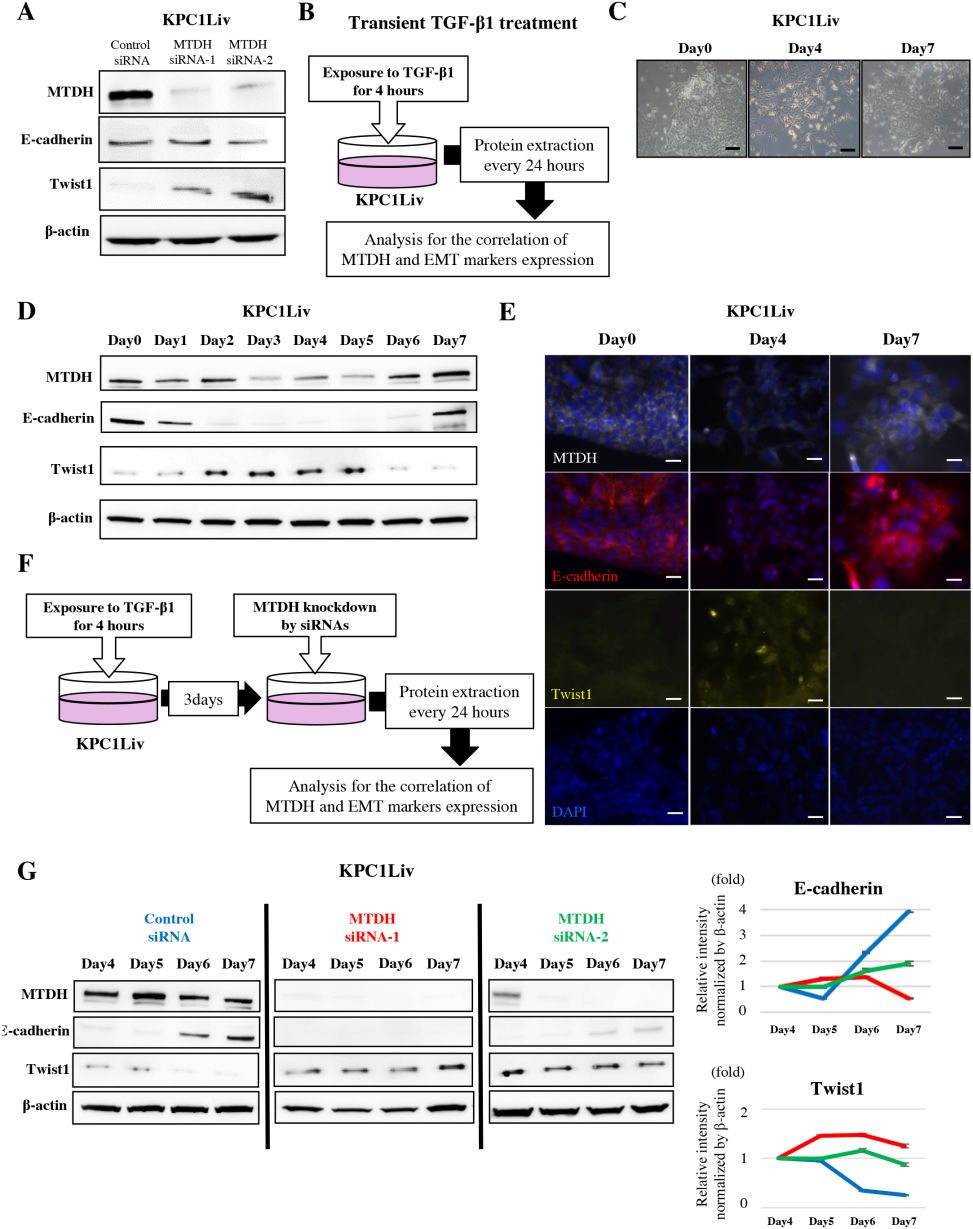
MTDH expression induces MET by downregulation of Twist1 expression in pancreatic metastatic cancer cells **(A)** MTDH expression is negatively correlated with Twist1 expression and does not correlate with E-cadherin expression in KPC1Liv cell lines. **(B)** Transient TGF-β1 treatment experimental design. (**C**) Morphological change in response to transient TGF-β1 treatment in KPC1Liv cells. **(D**) The expression pattern of E-cadherin, Twist1 and MTDH in response to transient TGF-β1 treatment in KPC1Liv cells (Western blotting). **(E**) Images of immunofluorescence staining for MTDH, E-cadherin, Twist, and DAPI in KPC1Liv cells at day 0, 4 and 7 after transient TGF-β1 treatment. Twist1 is highly expressed in the nucleus of cells at day 4 after treatment when both MTDH and E-cadherin are relatively suppressed. **(F)** Experimental set up to evaluate the correlation between MTDH and Twist1 at MET induction in KPC1Liv cells. **(G)** E-cadherin re-activation and Twist1 de-activation by transient TGF-β1 is attenuated by MTDH knockdown in KPC1Liv cells. The band intensities of E-cadherin and Twist1 were normalized by β-actin using densitometry analysis in right panels (Blue line: control siRNA, red line: MTDH siRNA-1 and green line: MTDH siRNA-2). Bar, 50μm.

Recent studies suggested that the repression of EMT or mesenchymal epithelial transition (MET) is one of the most crucial steps for metastatic colonization. To elucidate the molecular mechanism by which MTDH fosters PDAC metastatic colonization, we investigated the correlation between MTDH and E-cadherin or Twist1, a classical EMT marker in mouse PDAC metastatic cells. Unexpectedly, MTDH knockdown using siRNAs did not lead to a decrease of E-cadherin expression, but notably, increased Twist1 expression in KPC1Liv, metastatic mouse PDAC cells (Figure 3A).

To further explore how MTDH is correlated with E-cadherin and Twist1 during the EMT/MET process, we evaluated these expression patterns in response to transient TGF-β1 treatment in KPC1Liv cells (Figure 3B). After transient TGF-β1 treatment, we first observed morphological changes in KPC1Liv cells primarily exhibiting epithelial property (Figure 3C). E-cadherin expression was attenuated from 48 hours after treatment (EMT initiation), and then re-expressed at 7 days after treatment (MET induction) in KPC1Liv cells (Figure 3D, Supplementary Figure 3). In contrast, Twist1 expression increased at EMT initiation and decreased at MET induction. Interestingly, MTDH expression was positively and negatively correlated with E-cadherin and Twist1, respectively, during the process of EMT-MET plasticity in PDAC cells (Figure 3D, Supplementary Figure 3). These findings were also confirmed by immunofluorescence staining for MTDH, Twist1 and E-cadherin in KPC1Liv cells (Figure 3E).

To validate the correlation between MTDH and Twist1, Twist1 expression at MET induction was examined in MTDH-knocked down KPC1Liv cells in which EMT was induced by transient TGF-β1 treatment (Figure 3F). After EMT induction by TGF-β1, Twist1 expression was maintained even at the late period of MET induction, and E-cadherin was continuously suppressed by MTDH knockdown in KPC1Liv cells (Figure 3G). These results suggest that MTDH might induce MET by downregulation of Twist1 expression in PDAC cells.

### MTDH expression is associated with hematogenous dissemination and poor prognosis of patients with PDAC

**Figure 4:**
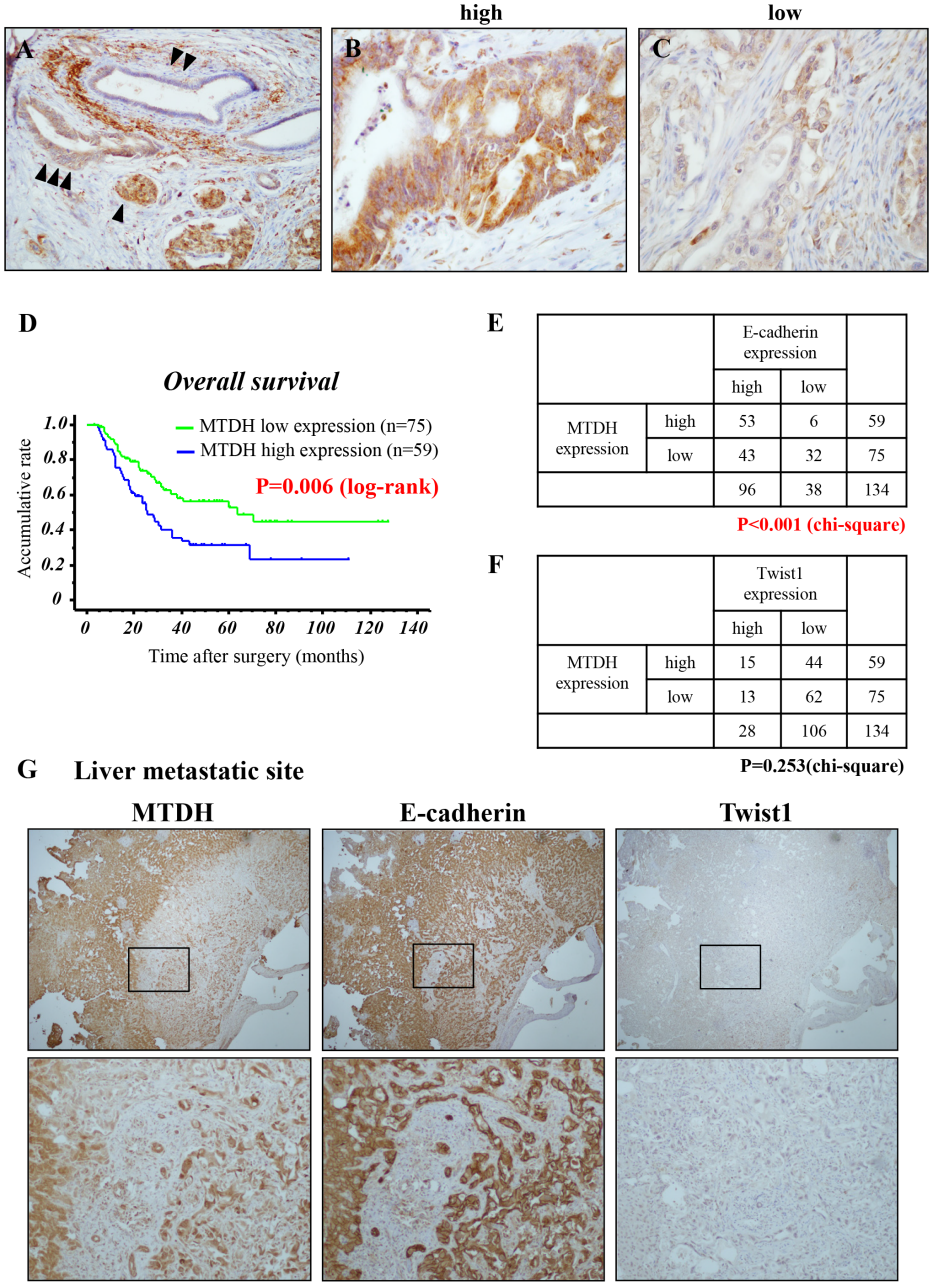
MTDH is expressed in the membrane of PDAC and high MTDH expression predicts poor prognosis in patients with PDAC after curative resection **(A-C)** Representative immunohistochemical staining of MTDH in primary PDAC tissues. (A) Islet cells (internal positive control) (▲), normal pancreatic duct (▲ ▲), PDAC (▲ ▲ ▲). (B) High MTDH expression in primary PDAC. (C) Low MTDH expression in primary PDAC. **(D)** Kaplan-Meier analysis for overall survival of patients with PDAC based on MTDH staining. Patients with high MTDH expression presented a significantly poorer prognosis than patients with low MTDH expression after curative surgery (*P* = 0.006: log-rank test). **(E, F)** The correlations between MTDH expression and E-cadherin expression (E) or Twist1 expression (F) in primary PDAC tissues. (chi-square test). **(G)** Representative staining for MTDH, E-cadherin and Twist1 in liver metastasis of PDAC tissues (Upper panels: low magnification: x100, Lower panels: high magnification: x200).

Finally, to assess the correlation between MTDH expression and the clinicopathological parameters in PDAC, we first evaluated MTDH expression in PDAC tissues by immunohistochemistry (IHC) (Table 1). To avoid surgical bias, we investigated PDAC cases with negative pathological surgical margin (R0). Whereas MTDH is slightly expressed in normal pancreatic duct, it is strongly expressed the membrane and cytoplasm of cancer cells (Figure 4A). All tissue samples were categorized into two groups (high: Figure 4B and low: Figure 4C) for MTDH according to the staining index (see Materials and Methods). Fifty-nine of all 134 cases (44.0%) were defined as MTDH high expression and 75 cases (56.0%) as MTDH low expression. High MTDH expression was significantly associated with UICC-T stage (*P* = 0.02, chi-square test) and hematogenous dissemination after surgery (*P* = 0.02, chi-square test; Table 1). The Kaplan-Meier analysis showed that patients with high MTDH expression presented a significantly poorer prognosis than those with low MTDH expression (*P* = 0.006, log-rank test; Figure 1E). On univariate analysis, T stage, N stage, histological grade, and MTDH expression were correlated with overall survival of patients with PDAC. Furthermore, among these factors, multivariate analyses indicated that high MTDH expression was an independent prognostic factor. (*P* = 0.005, cox’s proportional hazards model; Table 2). These clinical data suggests that high MTDH expression is associated with hematogenous dissemination and poor prognosis in patients with PDAC.

**Table 1:** Characteristics of PDAC patients in IHC analysis for MTDH expression

	MTDH expression		*P* value
	high (n=59)	low (n=75)	
Age (mean±SD)	66.2±9.7	66.8±9.4	NS
Female	21	32	NS
Male	38	43	
UICC			
Stage IIA ≥	19	25	NS
Stage IIB ≤	40	50	
pT1,2	0	7	*P* = 0.02
pT3,4	59	68	
pN0	20	26	NS
pN1	39	49	
Histological grade			NS
Well and Moderately	53	62	
poorly	6	13	
Local recurrence			
+	20	34	NS
-	39	41	
Hematogenous recurrence			
+	34	28	*P* = 0.02
-	25	47	

(chi-square test).

p: pathological findings, T: primary tumor, N: regional lymph nodes, NS: not significant.

**Table 2:** Univariate and multivariate analyses for overall survival of patients

	n=134	Univariate analysis Hazard ratio (95% CI)	*P* value	Multivariate analysis Hazard ratio (95% CI)	*P* value
Age (years) (≥67/≤66)	(67/67)	1.361(0.839-2.208)	0.815		
Gender (Female/male)	(53/81)	0.854(0.518-1.408)	0.869		
Histological grade (Poorly/others)	(19/115)	**1.988(1.035-3.817)**	**0.039**	1.923(0.972-3.802)	0.060
pT3,4/ pT1,2	(127/7)	6.536(0.907-47.619)	0.063		
pN1/ pN0	(88/46)	**2.519(1.416-4.484)**	**0.002**	**8.929(1.706-47.619)**	**0.010**
Stage IIB≤/≤IIA	(90/44)	**2.058(1.172-3.623)**	**0.012**	3.906(0.781-19.540)	0.097
Metadherin expression (high/low)	(59/75)	**1.941(1.196-3.150)**	**0.007**	**2.023(1.241-3.298)**	**0.005**

(Cox’s proportional hazard model).

p: pathological findings, T: primary tumor, N: regional lymph nodes, NS: not significant. CI: confidence interval.

Next, we estimated E-cadherin and Twist1 expression in resected human PDAC tissues by IHC staining to validate the correlation between MTDH and these expressions in PDAC. Interestingly, although there is no correlation with MTDH and Twist1, MTDH were strongly correlated with E-cadherin in primary PDAC site (Figure 4E and 4F). Consistent with this, we obsereved a similar expression pattern between MTDH and E-cadherin in liver metastatic site (Figure 4G). Taken together, these results suggested that MTDH is closely associated with epithelial phenotype during PDAC progression.

## DISCUSSION

**Figure 5:**
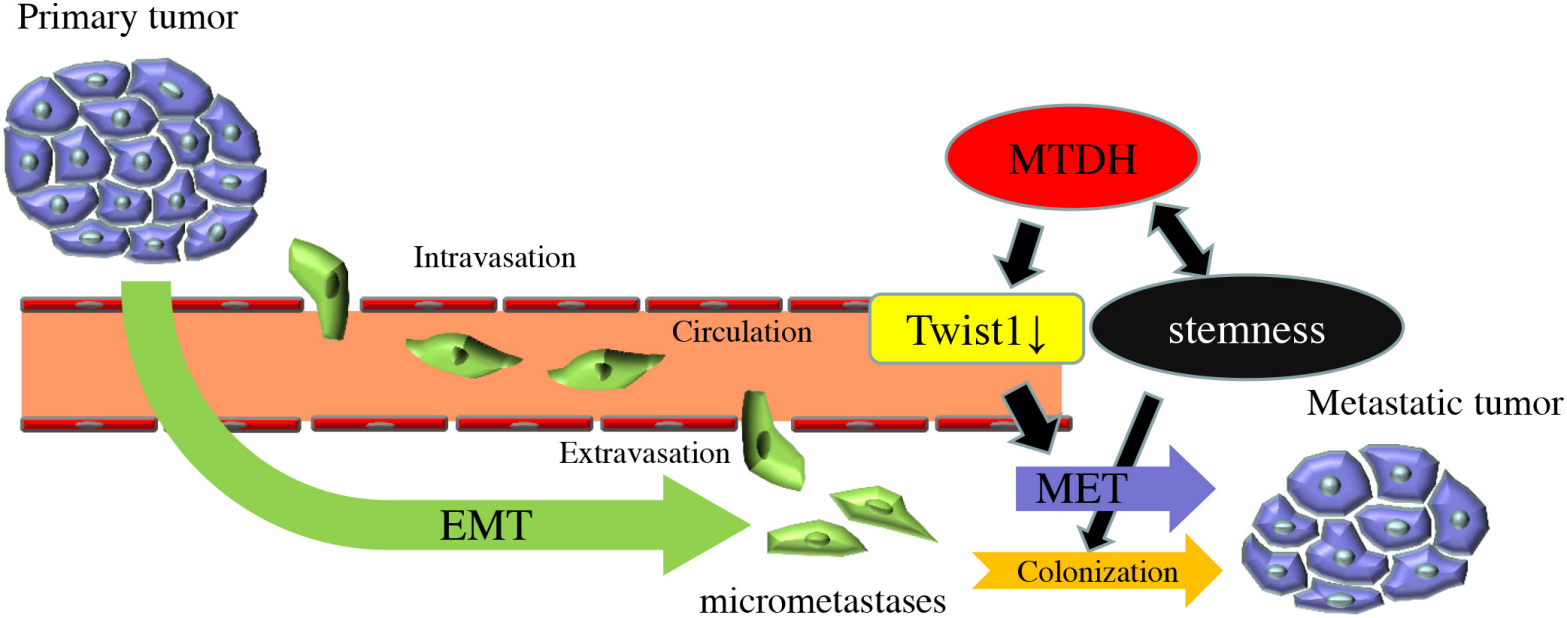
Model of the functional roles of MTDH in PDAC metastatic progression

In this study, we demonstrated that MTDH promotes CSC property of PDAC cells and prevents disseminating cancer cells from anoikis. Furthermore, MTDH is associated with the suppression of Twist1 expression, supporting the switch of tumor phenotype from mesenchymal to epithelial cells to accelerate metastatic colonization with epithelial plasticity. The analysis of human clinical samples indicated that MTDH is significantly associated with hematogenous dissemination, resulting in poor prognosis of patients with PDAC. Our results reveal a novel mechanistic insight of MTDH, which is functionally correlated with epithelial plasticity in metastatic cascade of PDAC (Figure 5).

MTDH was initially reported as a protein mediating metastasis of mouse breast cancer [12]. Further functional investigations revealed that MTDH also mediates tumor progression, proliferation, angiogenesis, invasiveness, and metastasis [9, 13, 16, 17]. Numerous studies support the notion that EMT and CSC properties are responsible for metastasis, and EMT also endows cancer cells with CSC properties [6, 18]. Previous experimental data indicated that MTDH regulates EMT and promotes CSC accumulation in breast cancer [19]. In line with these data, we demonstrated that MTDH is associated with the enhancement of self-renewal capacity and the expression of a CSC surface marker in PDAC cells. Specifically, MTDH significantly fostered CSC properties in metastatic PDAC cells, but not in primary PDAC cells, suggesting that MTDH might be required for the promotion of CSC properties at the metastatic site.

Meanwhile, we demonstrated that MTDH is positively correlated with MET in metastatic PDAC. Notably, some groups recently revealed that EMT and CSCs properties are not always inextricably linked [20], and that the plasticity of sequential EMT and MET allows cancer cells to acquire CSCs capacity for an efficient metastatic colonisation [21, 22]. Twist1, known as a classical EMT marker, has recently garnered the attention as one of the key molecules for the elucidation of the mechanisms that regulate MET [23]. Our data using transient TGF-β1 stimulation suggested that MTDH is required for the suppression of Twist1, thereby supporting MET in PDAC cells. Consistently, several experimental systems revealed that transient activation of Twist1 induces MET and CSC properties [22, 24]. Furthermore, Fabregat *et al.* proposed that EMT contributes to the acquisition of CSCs, even though turning off Twist1, key master regulation of EMT, is necessary to acquire CSCs at the metastatic site [25]. Further elucidation for this molecular mechanism between MTDH and Twist1 is clearly warranted.

Liang *et al.* demonstrated that MTDH regulates EMT and contributes the promotion of CSC properties in invasion (EMT phase) in breast cancer progression [19]. Contrary to this, we revealed that MTDH induces stemness in metastatic colonization (MET phase) in PDAC progression. Indeed, the biological behavior of PDAC is different from that of breast cancer, especially in the property of invasiveness. It might be possible that the types of phenotypic switch (EMT or MET) with CSC properties regulated by MTDH are primarily dependent on cancer types. We described that the function of MTDH related to CSC properties regulates epithelial plasticity for the period of metastatic colonization in PDAC.

In addition, we identified Prrx1 as an overlapping key transcription factor among pancreatic development, regeneration and PDAC progression [26]. Prrx1a and Prrx1b, two major isoforms of Prrx1, play functionally opposing roles in PDAC progression, Prrx1a contributes to the promotion of MET and the acquisition of stem-like property for metastatic colonisation during PDAC progression [27]. Interestingly, ChIP sequence analysis indicated that MTDH is one of the downstream targets of Prrx1a. These results imply that MTDH might play a crucial role in the regulation of epithelial plasticity with CSCs properties to drive delaminating PDAC cells to form overt metastatic colonies. It is tempting to speculate that MTDH is regulated by Prrx1a to establish the metastatic colonization, a subject of future investigation.

We next examined the last step of the metastatic cascade (colony formation after extravasation from blood vessels) in *in vitro* studies, anoikis assay was utilized to evaluate both resistance to apoptosis after losing contact with the extracellular matrix and subsequent colonisation. *In vivo* experiments, the functional roles of MTDH in PDAC metastatic site was assessed in the intra-portal vein injection model created to exclude the invasion process from the pancreas into the systemic circulation. Our results in both experimental models indicate the crucial role for MTDH in the colonisation at the distant site.

In this study, we demonstrated that high MTDH expression is associated with unfavorable outcome and is an independent prognostic factor in patients with PDAC. Previous reports also described that high MTDH expression is closely linked to poor prognosis in breast, hepatocellular, esophageal and colorectal cancers [9, 14, 28, 29]. Moreover, we determined that MTDH and E-cadherin were strongly correlated by IHC staining of resected human PDAC tissues. This finding is contrary to previous reports [29, 30]. This discrepancy might be due to the fact that the biological function of MTDH in PDAC is different from that in other cancers. Indeed, recent findings proposed that epithelial plasticity is crucial for cancer progression, especially metastatic colonization in distant organ [31]. Thus, the function of MTDH for epithelial plasticity might enhance the metastatic potential in PDAC.

In conclusion, we demonstrated for the first time that MTDH promotes metastasis by supporting CSC properties and epithelial plasticity in PDAC. Our experimental data suggest that MTDH facilitates MET, in part, by downregulation of Twist1 in metastatic PDAC cells. This study presents some limitations. We only analyzed the role of MTDH by knockdown in order to investigate the cellular response in physiological conditions. Further MTDH gain of function experiments are required to elucidate the correlation between MTDH and Twist1 during metastatic colonization. MTDH might be a novel target for the regulation of metastasis in PDAC. Controlling epithelial plasticity by modulating MTDH during PDAC progression may provide a new strategy for the treatment of PDAC.

## MATERIALS AND METHODS

### Patient samples

PDAC tissues were obtained from 134 consecutive patients who underwent microscopically curative surgical resection (R0) at the Department of General Surgery, Chiba University Hospital, Japan, from June 2006 to March 2015. All patients were histologically diagnosed with primary invasive PDAC. The patients’ characteristics are described in Table 1. The study protocol has been approved by the Ethics Committees of our institute and written informed consent was obtained from each patient before surgery.

### Human and murine cell lines

The human pancreatic cell lines, PANC-1, MIA PaCa-2, Capan-1, AsPC-1, CFPAC-1, and Hs766T, were obtained from the American Type Culture Collection (Manassas, VA, U.S.A) The PANC-1 and MIA PaCa-2 in Dulbecco’s Modified Eagle Medium (DMEM: Sigma-Aldrich, St Louis, MO, USA) with 10% fetal bovine serum (FBS) and antibiotics (1% penicillin and streptomycin), CFPAC-1 and Capan-2 in Iscove’s Modified Dulbecco’s Medium (IMDM: Thermo Fisher Scientific, Waltham, MA, USA) with 10% FBS and antibiotics, and AsPC-1 cells in RPMI-1640 medium (Thermo Fisher Scientific) with 10% FBS and antibiotics. Mouse primary pancreatic cells were cultured and maintained as described previously [26]. Murine PDAC (KPC1) and paired metastases (KPC1Liv) cell lines were provided by Dr. Sunil Hingorani (University of Washington). In brief, KPC1 cell lines were established from primary PDAC of a genetically engineered mouse model of PDAC (*LSL-Kras*^*G12D/+*^*;p53*^*R172H/+*^*;Pdx1-cre*) [32], whereas the KPC1Liv cell lines were isolated from paired liver metastases arising in KPC1 mice. KPCY cells are derived from a *Pdx1-cre;LSL-Kras*^*G12D/+;p53fl/+*^*;R26*^*YFP*^ mouse (KPCY mice) and were provided by Dr. Andrew D. Rhim (The University of Texas MD Anderson Cancer Center).

### Immunohistochemical and immunofluorescence staining

Immunohistochemical staining of MTDH was performed following standard protocols. Briefly, paraffin-embedded tissue blocks were cut into 4-µm thick sections. The tissue sections were incubated with anti-MTDH antibodies (Cat#40-6500, Invitrogen, Carlsbad, CA, USA; dilution 1:400) overnight at 4°C, incubated in Envision^TM+Kits^ (Dako, Glostrup, Denmark), and visualized using 0.01% 3, 3-diaminobenzidine. The staining patterns were scored as follows: low expression: 0–50% of tumor cells with positive staining; high expression: more than 50% of tumor cells with positive staining. The intensity of staining of islet cells was used as an internal positive control. The score of immunohistochemical staining was evaluated independently by three investigators. Immunofluorescence staining was carried out as described previously [26]. The following primary antibodies are used in this study: anti-MTDH (Invitrogen; 1:50), anti-E-cadherin (Cat#610182, BD Biosciences, Franklin Lakes, NJ, USA; 1:50), anti-Twist1 (Cat#sc-81417, Santa Cruz, Dallas, TX, USA; 1:50), anti-GFP (Cat#ab13970, Abcam, Cambridge, UK; 1:250).

### RNAi transfection and vector constructs

MTDH siRNAs (MTDH siRNA1: Cat#S100007602, MTDH siRNA2: Cat#S100007609) and control siRNA (AllStars negative control siRNA) were purchased from QIAGEN (Hilden, Germany). Cells (4.0x10^5^) were plated in a 60-mm dish and incubated for 24 hours. Cells were then treated with siRNA (10 nM final concentration) in Lipofectamine^TM^ RNAiMAX Transfection Reagent (Invitrogen). Cells were detached and re-plated for the cell cytotoxic assay after 24 hours of siRNA treatment. Protein was harvested from cells after culturing for 72 hours. Lentiviral transduction was performed as previously described [26]. Lentiviral vectors were purchased from Sigma-Aldrich. (MTDH shRNA-1: Product type: SHCLNV-NM_026002, The RNAi Consortium Number: TRCN0000312351, MTDH shRNA-2: Product type: SHCLNV-NM_026002, TRC Number: TRCN0000125816, or control shRNA: Product Number: SHC016V-1EA).

### Quantitative real-time PCR

Total RNA from each pancreatic cancer cell line was purified using RNeasy Mini Kit (QIAGEN) according to the manufacturer’s instructions. Complementary DNA (cDNA) was synthesized from mRNA using SuperScript VILO cDNA Synthesis Kit and Master Mix (Life Technologies, Palo Alto, CA, USA). Quantitative real-time reverse transcriptase-polymerase chain reaction (RT-PCR) was performed with the Sequence Detection System ABI Prism 7300 (Applied Biosystems, Foster City, CA, USA), using the SYBR Green method with following reagents: MTDH primers (hMTDH-Forward: 5’-CTTGGTCCCACTAGCCATGAA-3’, hMTDH-Reverse: 5’-GACACTGTTAGAGGTGCCCATTATC-3’, from TAKARA BIO INC., Kusatsu, Shiga, Japan) and FAST SYBR Green PCR Master MIX (Life Technologies).

### Western blot analysis

Total protein was purified from cultured cells with Radio-Immunoprecipitation Assay (RIPA) Buffer (SIGMA-ALDRICH) and preserved at-80°C. Thirty micrograms of protein were loaded onto a 7.5-15% XV PANTERA Gel (DRC, Tama, Tokyo, Japan), and transferred onto a polyvinylidene difluoride (PVDF) membrane. The membranes were blocked at room temperature for 60 minutes in 5% milk in 0.1% Tris Buffed Saline with Tween-20 (TBS-T). Membranes were incubated with anti-MTDH (Invitrogen; 1:1000), anti-E-cadherin (Cat#sc-7870, Santa Cruz; 1:1000), anti-Twist1 (Santa Cruz; 1:300), and anti-β-actin (Cat#13E5, Cell Signaling, Danvers MA, USA; 1:2000) antibodies overnight at 4°C, washed three times for 5 minutes each with TBS-T, and incubated with secondary antibodies (anti-rabbit IgG horseradish peroxidase; Santa Cruz Biotechnology; 1:3000). The membranes were developed using ImageQuant LAS-4000UV mini Mac (General Electoric Company, Fairfield, CT, USA) after immersion in the detection reagent. Western blots were quantified by densitometry analysis and normalized to β-actin using the Image J software.

### Pancreatosphere formation assay

Pancreatosphere formation assays were performed as described previously [33]. In brief, cells with or without siRNA treatment were seeded in 96-well ultra-low attachment plates (Corning, New York, USA) at a density of 10 cells per well. Cells were grown for seven days in sphere medium. We defined a cell cluster with over 50 µm diameters on day 7 as a sphere. The sphere formation rate was calculated by the number of spheres on day 7 divided by the number of cell clusters on day 1.

### Flow cytometry analysis

Two million cells were suspended in 100 µL PBS and incubated with antibodies for 60 minutes on ice in the dark. Antibodies used were; APC anti-human CD133 (Miltenyi Biotec, Bergisch gladbach, Germany; 1:100), APC anti-mouse CD133 (Prominin-1) (Miltenyi Biotec; 1:50), APC anti-CD326 (EpCAM) (Miltenyi Biotec; 1:100). After washing with PBS, cells were re-suspended in 1 mL PBS and measured by a CANTO II system (Beckton-Dickinson). All Data were analyzed using FlowJo v10.1r5 software (Ashland, OR, USA).

### Three-dimensional cell culture

Three-dimensional (3D) pancreatic ductal cell culture was performed as previously described [34, 35]. In brief, Collagen I solution was spread onto 4-well chamber slides (Thermo Fischer Scientific) as a bottom layer and set in 37°C oven for 30-60 min. Control and MTDH knockdown KPCY cells seeded in plates were trypsinised and suspended in Collagen I solution. This gel-cell solution was added to well plates on the bottom layer and placed in 37°C oven for 60 min to allow gelation, after adding culture medium to each well on this gel-cell solution. After keeping them in the incubator at 37°C for 10 days, cells were imaged using the AxioVision (version 4.3; Carl Zeiss, Oberkochen, Germany) and spheroid cysts were counted.

### Anoikis assay

To evaluate anoikis resistance (the resistance for apoptosis after losing contact with the extracellular matrix), 2000 KPCY cells/ml [4] infected with lentivirus (MTDH shRNA-1, MTDH shRNA-2 or control shRNA) were incubated with medium without growth factor with rotation for 24 hours at 37°C. Next, colony formation assay was performed (see details in the previous report [27]). The number of colonies was determined at 21 days after cell seeding.

### Orthotopic transplantation/intra-portal injection models

The *in vivo* metastasis assay was conducted as previously described [27]. In the orthotopic transplantation model, female nude KSN/slc mice (Japan SLC Inc., Hamamatsu, Japan) at the age of 9 weeks were injected with 5 × 10^4^ KPCY cells infected with lentiviral constructs (MTDH shRNA-1, MTDH shRNA-2, or control shRNA) into the pancreas tails under anesthesia. At 14 days after cell injection, the mice were euthanized and primary pancreatic tumor and the whole liver were removed. The volume of primary tumor was measured, and serial sections were made from the liver every 1000μm. Liver metastases were counted under the microscope.

In intra-portal vein injection model, female nude KSN/slc mice at the age of 9 weeks were injected with 1 × 10^5^ KPCY cells infected with lentivirus into the portal vein under anesthesia. After 14 days, the mice were euthanized and the whole liver were removed. Liver metastases were counted as previously described [27]. Animal studies were approved by the Committee on the Use of Live Animals for Teaching and Research of the Chiba University.

### Transient TGF-β1 treatment

Transient TGF-β1 treatment was performed as described previously [23]. In brief, proteins were extracted from KPC1Liv cells cultured for 24 hours after exposure to 4 ng/ml TGF-β1 for 4 hours, and the expression pattern of MTDH and EMT markers was investigated by western blotting.

### Statistical analysis

All statistical analyses were conducted with the Statview-J5.0 software package (SAS Institute Inc., Cary, NC, USA). Accumulative rates were calculated by using the Kaplan-Meier method and the significance of difference in survival rate was analyzed by the log-rank test. Data are expressed as mean ± SD. Survival data were evaluated using univariate and multivariate Cox proportional regression analyses. Statistically significant differences were determined by Student’s t-test, Chi-square test, or Fisher’s exact test. *P* < 0.05 was considered significant in all analyses. Each experiment was repeated at least three times in triplicate.

## SUPPLEMENTARY MATERIALS FIGURES


