# Supplementary material for: Metadherin promotes metastasis by supporting putative cancer stem cell properties and epithelial plasticity in pancreatic cancer
Source: Oncotarget. 2017 Aug 2;8(39):66098–111. doi: 10.18632/oncotarget.19802 (PMC5630395; doi:10.18632/oncotarget.19802)
Supplement: Supplementary file 1 [file oncotarget-08-66098-s001.pdf]

Metadherin promotes metastasis by supporting putative cancer stem cell properties and epithelial plasticity in pancreatic cancer

SUPPLEMENTARY MATERIALS

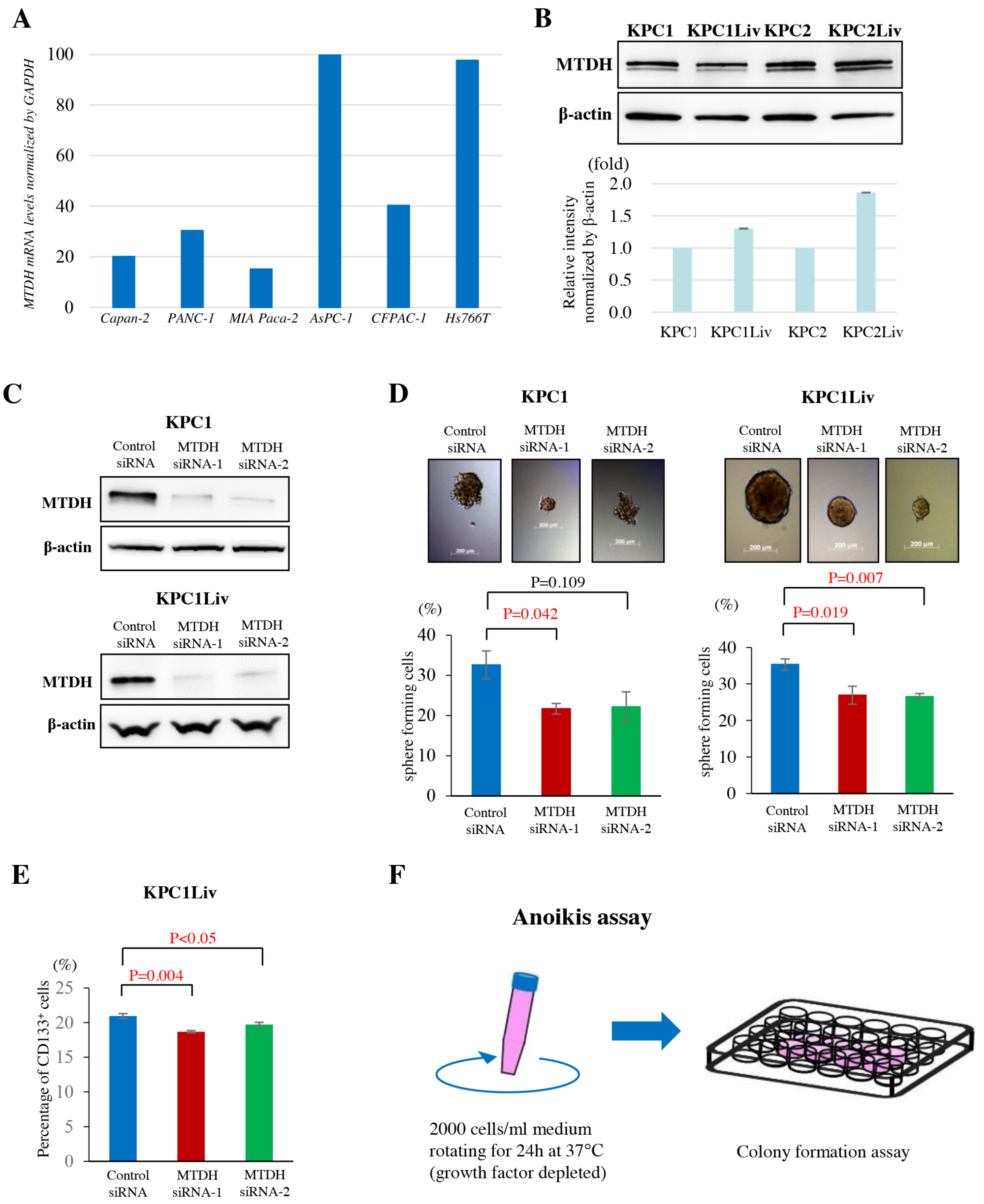

Supplementary Figure 1: **(A)** MTDH mRNA levels in metastatic PDAC highly expressed compared to primary PDAC cell lines by quantitative RT-PCR. **(B)** Comparison of MTDH protein expression between mouse primary PDAC cells (KPC1 and KPC2) and liver metastatic PDAC cells (KPC1Liv and KPC2Liv cells) by Western blot analysis. Band intensities were normalized by densitometry to β-actin. **(C)** Knockdown of MTDH protein expression for KPC1 and KPC1Liv by MTDH siRNA-1 and siRNA-2 was confirmed by Western blot analysis. (**D**) The sphere formation rate in KPC1 and KPC1Liv cells treated with negative control siRNA, MTDH siRNA-1, or siRNA-2. Upper panel: Representative respective sphere forming cells. Lower panel: MTDH knockdown significantly decreases the sphere formation rate in KPC1Liv metastatic cells. (student's t-test). **(E)** Analysis for CD133 expression in KPC1Liv cells by flow cytometry. MTDH knockdown leads to decrease the proportion of $CD133^{+}$ cells. (student's t-test). Values are shown as mean ± SEM. **(F)** Experimental setup for anoikis assay.

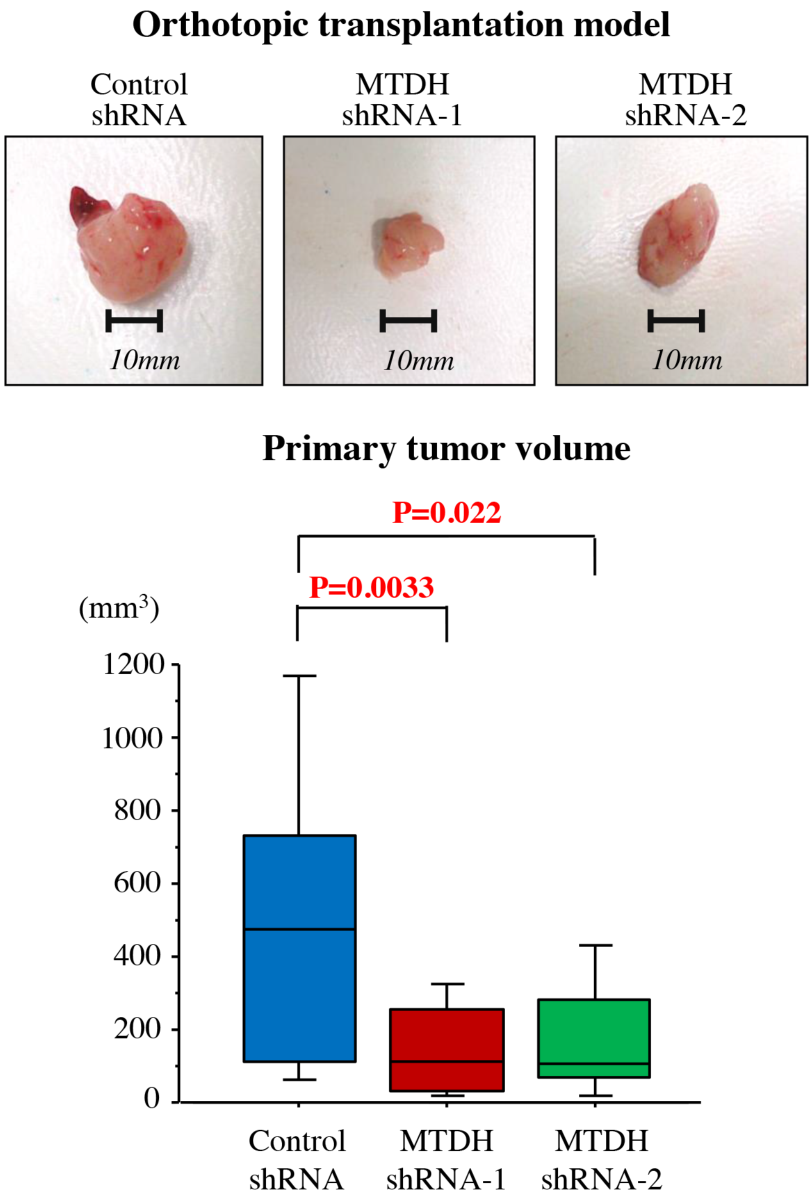

Supplementary Figure 2: Primary tumor volume in orthotopic transplantation mouse PDAC models. Primary tumor volume of KPCY-MTDH shRNA-1 group (n=14) and shRNA-2 group (n=14) established significantly smaller than that of KPCY-control shRNA group (n=14). The tumor volume was measured using a caliper and calculated using the following formula: $\pi/6 \times (L \times W \times W)$, where L is the tumor at its longest and W is at its shortest. Values showing box-plot histogram are indicated as mean ± SEM.

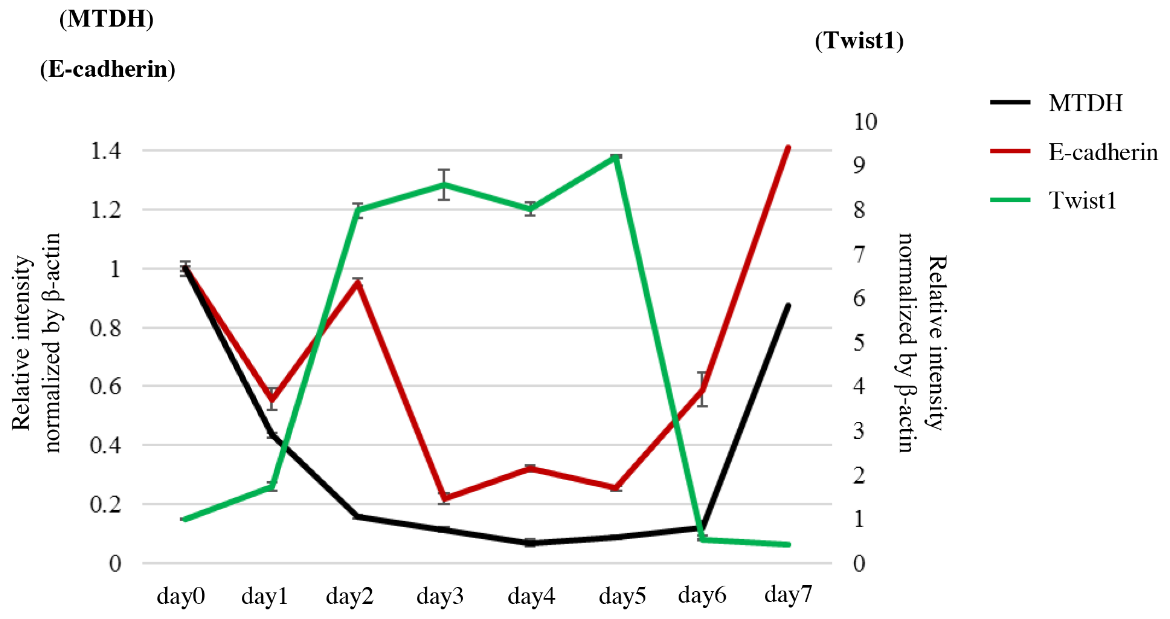

Supplementary Figure 3: Relative intensities of E-cadherin, Twist1 and MTDH expression in response to transient TGF-β1 treatment in KPC1Liv cells (Western blotting). Band intensities were normalized by densitometry to β-actin.
